# Silencing lncRNA AK136714 reduces endothelial cell damage and inhibits atherosclerosis

**DOI:** 10.18632/aging.203031

**Published:** 2021-05-18

**Authors:** Jing Bai, Jianxia Liu, Zexian Fu, Yuanyuan Feng, Bing Wang, Wenjuan Wu, Ruiying Zhang

**Affiliations:** 1 Department of Geriatrics, Affiliated Hospital of Hebei University of Engineering, Handan 056000, Hebei Province, China; 2 Department of Nursing, Affiliated Hospital of Hebei University of Engineering, Handan 056000, Hebei Province, China; 3 Department of Scientific Research and Education, Affiliated Hospital of Hebei University of Engineering, Handan 056000, Hebei Province, China; 4 Department of Stomatology, Affiliated Hospital of Hebei University of Engineering, Handan 056000, Hebei Province, China; 5 Department of Dynamic electrocardiogram, Affiliated Hospital of Hebei University of Engineering, Handan 056000, Hebei Province, China; 6 Department of Breast, The Affiliated Hospital of Hebei University of Engineering, Handan 056000, Hebei Province, China

**Keywords:** lncRNA, atherosclerosis, endothelial cells, AK136714, FOXO3

## Abstract

Atherosclerosis correlates with ischemic cardio-cerebrovascular diseases such as coronary heart disease. Long non-coding RNAs (lncRNAs) can promote atherosclerosis. We investigated the role of the lncRNA AK136714 in atherosclerosis. Compared with the healthy group, lncRNA AK136714 expression was elevated in the plaque and plasma of the atherosclerosis patients in a GEO dataset. AK136714 silencing inhibited atherosclerosis formation in ApoE-/- mice. AK136714 silencing also protected the endothelial barrier and inhibited endothelial cell inflammation. *In vitro* assays showed that knockdown of AK136714 suppressed the inflammatory response and apoptosis in human umbilical vein endothelial cells (HUVECs). Moreover, AK136714 was found to bind directly to HuR to increase the mRNA stability of TNF-α, IL-1β and IL-6 mRNAs. In addition, AK136714 promoted the transcription of Bim. This study expands our understanding of the role of lncRNA AK136714 in atherosclerosis and provides potential drug targets for the treatment of atherosclerosis.

## INTRODUCTION

Atherosclerosis is a chronic inflammatory disease that promotes ischemic cardiovascular and cerebrovascular disease [1–3]. Multiple atherosclerotic factors increase inflammation in the pathogenesis of atherosclerosis [4–6]. Long non-coding RNA (lncRNA) is linear RNA that is longer than 200 nucleotides and does not encode proteins [7, 8]. Some lncRNAs accelerate atherosclerosis pathogenesis by promoting endothelial cell dysfunction, vascular smooth muscle cell proliferation and migration, lipid metabolism disorders, and inflammatory responses [9–11].

FOXO3, a member of the forkhead box protein family, induces extracellular matrix breakdown and apoptosis in vascular smooth muscle, endothelial cells, and cardiomyocytes. FOXO3 also suppresses the effects of oxidative stress [12, 13]. HuR, also known as ELAVL1, is a widely expressed RNA-binding protein that stabilizes its mRNA targets and may promote atherosclerosis occurrence and development [14, 15]. We investigated the effects of lncRNA AK136714 on atherosclerosis. AK136714 can directly bind HuR and FOXO3 to inhibit inflammatory factor release and endothelial cell apoptosis.

## RESULTS

### AK136714 was increased in the plaque tissue and blood of atherosclerotic mice

**Figure 1:**
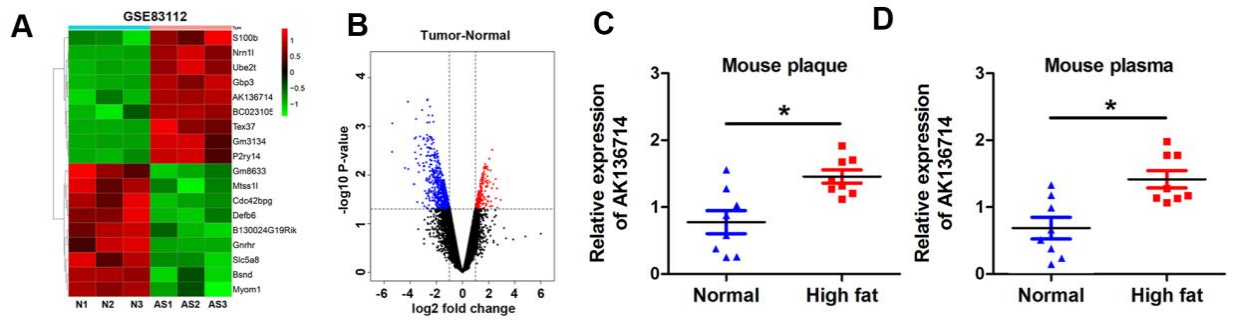
**AK136714 is reduced in plaque tissue and blood of atherosclerotic mice.** (**A**) GEO database analysis identified dysregulated lncRNAs in plaques from a mouse atherosclerosis model. (**B**) Volcano map showing the gene distribution. (**C**) Detection of AK136714 expression in plaques of atherosclerotic mice using qPCR. (**D**) Detection of AK136714 expression in the blood of atherosclerotic mice using qPCR. (n=8, *P < 0.05).

We first analyzed the differentially expressed lncRNA of mice on a normal diet and mice on a high-fat diet. By analyzing the GEO database, the change of lncRNA expression in atherosclerotic plaques was obtained in a mouse atherosclerosis model, and the heat map showed the expression of lncRNA in each group (Figure 1A). The volcano plot shows the gene distribution (Figure 1B). We found that AK136714 expression was increased in plaques in atherosclerotic mice compared to normal diet mice (Figure 1C). Upregulation of AK136714 expression was also detected in the blood of high-fat diet mice (Figure 1D).

### Silencing AK136714 inhibits atherosclerotic plaque formation

**Figure 2:**
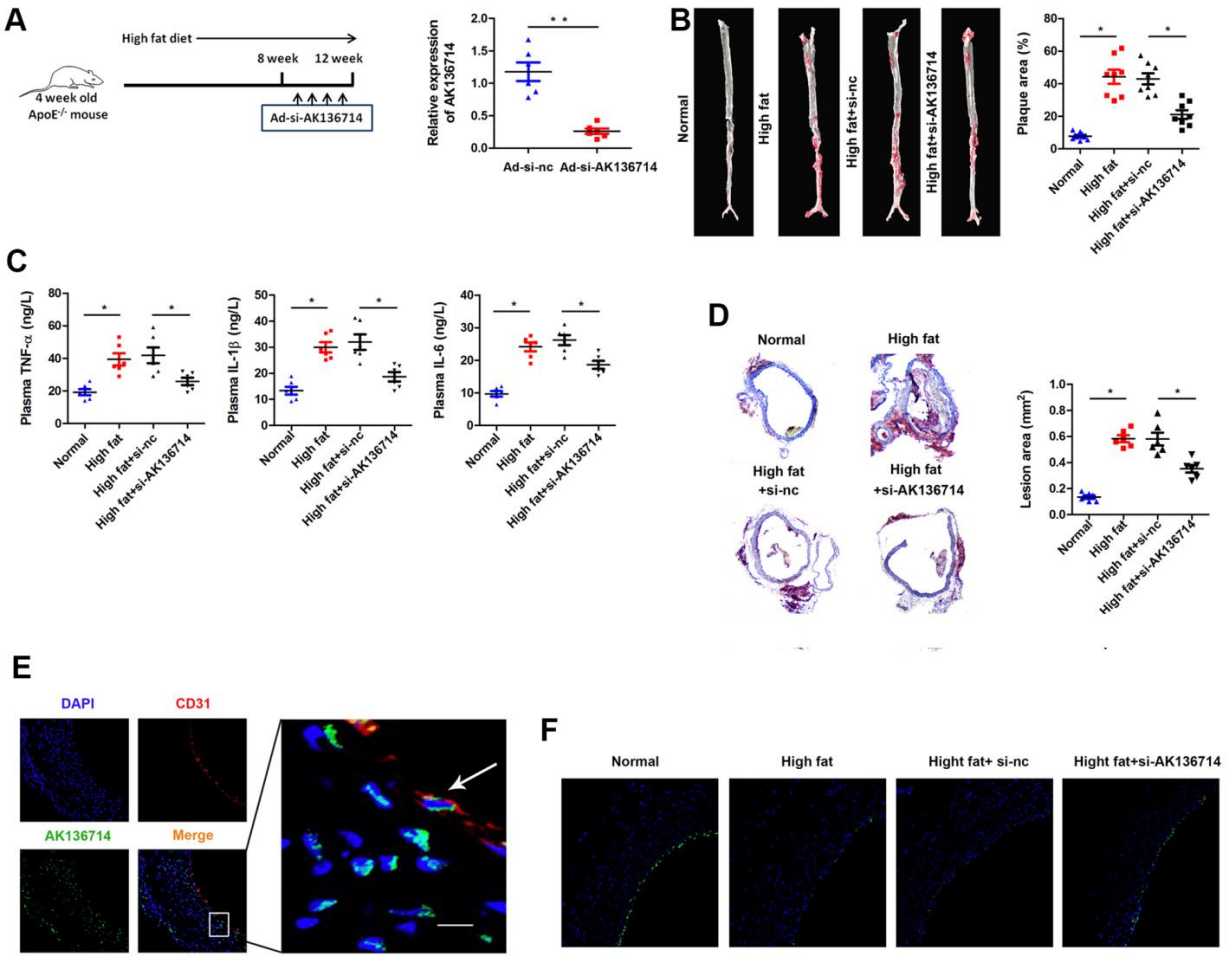
**Overexpression of AK136714 inhibits atherosclerosis.** (**A**) qPCR was used to detect the expression of AK136714 in vascular tissue of each group. (**B**) Vascular oil red O staining was performed to detect the degree of atherosclerosis. (**C**) ELISA was used to assess inflammatory factor expression. (**D**) Oil red O staining was performed to detect the degree of vascular injury. (**E**) Immunofluorescence detection showed that AK136714 is mainly in the cytoplasm of endothelial cells and colocalizes with the endothelial marker CD31. (**F**) Immunofluorescence staining of CD31 showed that high fat damages endothelial cells, and AK136714 knockdown can reverse endothelial cell damage. (n = 8, *P < 0.05, **P < 0.01).

We established an animal model of atherosclerosis using AK136714 knockdown by Adenovirus (Ad-si-AK136714) infection. High-fat feeding was continued for 12 weeks, and the adenovirus was injected into the tail vein once a week for 8-12 weeks. Using qPCR, AK136714 expression was detected in vascular tissues of each group, which showed that Ad-si-AK136714 could effectively decrease AK136714 expression (Figure 2A). When compared to the control group, vascular oil red O staining showed that atherosclerosis-induced plaque formation was diminished by silencing AK136714 (Figure 2B). ELISA testing showed that, compared with the control group, the production and release of inflammatory factors was increased in mice on a high-fat diet. Additionally, silencing AK136714 inhibited the release of inflammatory factors (Figure 2C). The degree of vascular injury detected by oil red O staining showed that compared with the control group, arterial injury was increased in high-fat diet mice, and silencing AK136714 inhibited arterial injury (Figure 2D). Immunofluorescence showed that AK136714 was mainly in endothelial cell cytoplasm and co-localized with endothelial marker CD31 (Figure 2E). High lipids damaged endothelial cells, and AK136714 silencing could reverse the endothelial cell damage (Figure 2F).

### Silencing of AK136714 inhibits endothelial cell apoptosis

**Figure 3:**
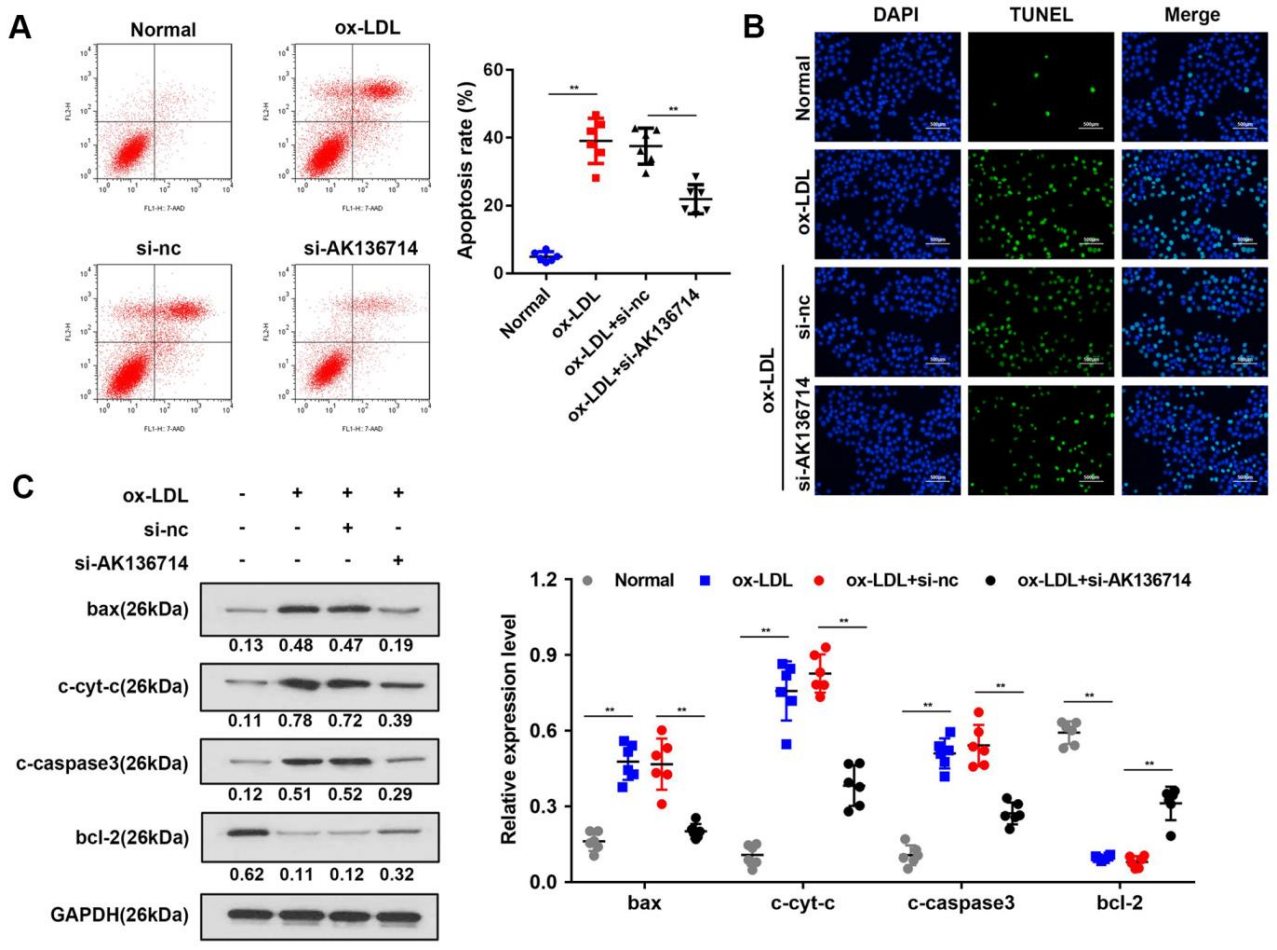
**Overexpression of AK136714 inhibits endothelial cell apoptosis.** (**A**) Flow cytometry was used to detect the apoptosis of vascular endothelial cells in ox-LDL-induced injury models. (**B**) TUNEL assay was performed to detect changes in apoptosis. (**C**) Western blot was used to detect the changes in expression of apoptosis-related proteins. (n=6, **P < 0.01).

Flow cytometry was used to detect vascular endothelial cell apoptosis in ox-LDL-induced injury models. Compared with the control group, ox-LDL treatment increased endothelial cell apoptosis, and silencing AK136714 inhibited endothelial cell apoptosis (Figure 3A). The TUNEL results were consistent with those of flow cytometry (Figure 3B). Western blot was used to detect the expression of apoptosis-related proteins. Compared with the control group, the expression of apoptotic proteins Bax, cytoplasmic cyt-c, and cleaved caspase3 increased in high-fat diet mice, while the expression of anti-apoptotic protein Bcl-2 decreased. Silencing of AK136714 inhibited the expression of bax, cytoplasmic cyt-c, cleaved caspase3 while promoted that of bcl-2 (Figure 3C).

### Silencing of AK136714 protects the endothelial barrier and inhibits the inflammatory response of endothelial cells

**Figure 4:**
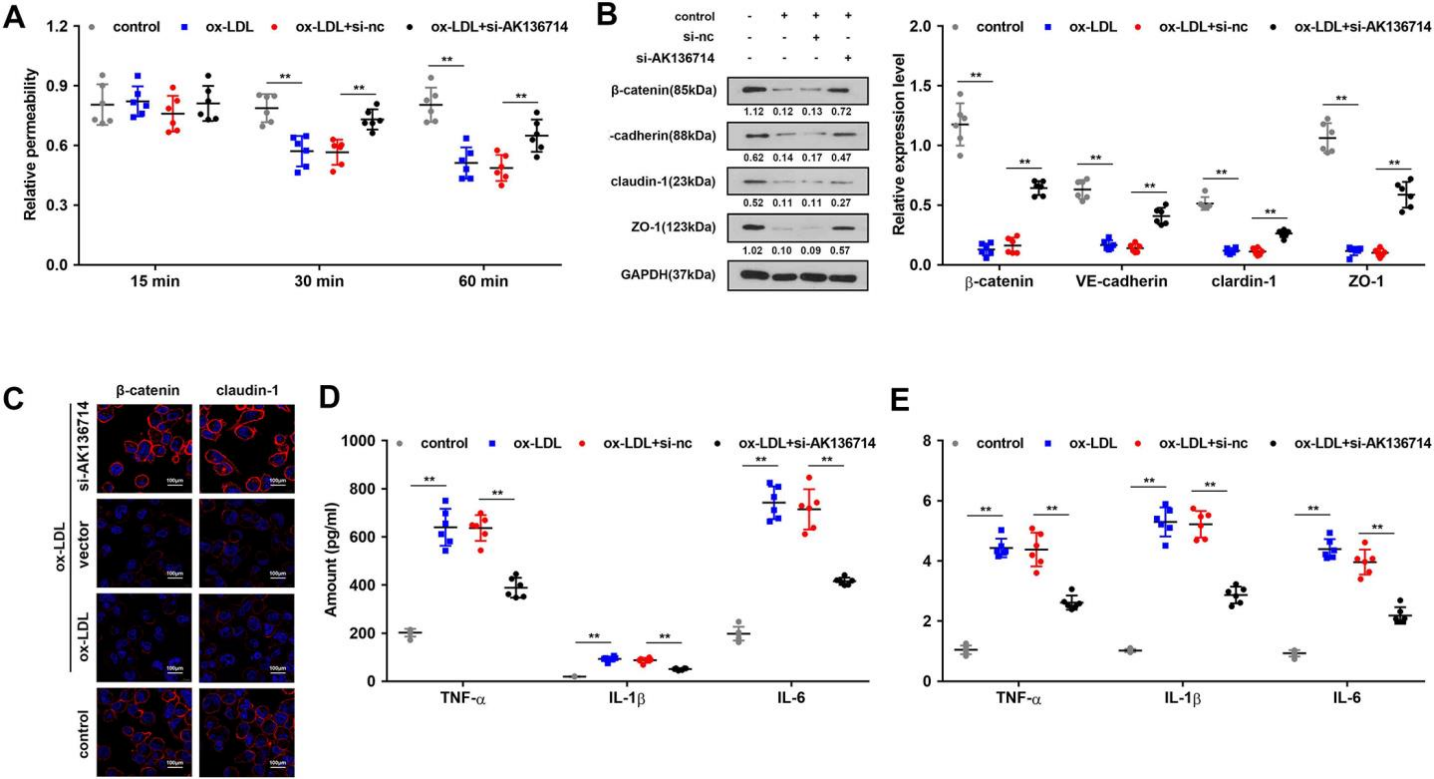
**Overexpression of AK136714 protects the endothelial barrier and inhibits the inflammatory response of endothelial cells.** (**A**) Detection of endothelial cell permeability. (**B**, **C**) Western blot and immunofluorescence were performed to detect endothelium tight junction biomarkers. (**D**) ELISA detection of inflammatory factor expression. (**E**) qPCR detection of inflammatory factor expression. (n=6, *P < 0.05).

Endothelial cell permeability experiments show that ox-LDL induction inhibits endothelial cell function and decreases endothelial cell permeability, while silencing of AK136714 can restore endothelial cell permeability (Figure 4A). Western blot and immunofluorescence detected endothelium tight junctions-related protein expression. In endothelial cells, ox-LDL inhibited tight junction proteins such as β-catenin, VE-cadherin, claudin-1 and ZO-1, while silencing of AK136714 restored the expression of those tight junction proteins (Figure 4B). ELISA and qPCR showed that ox-LDL promoted endothelial inflammatory factor levels, and silencing of AK136714 inhibited endothelial inflammatory factor levels (Figure 4D, 4E).

### AK136714 directly binds to HuR and FOXO3

**Figure 5:**
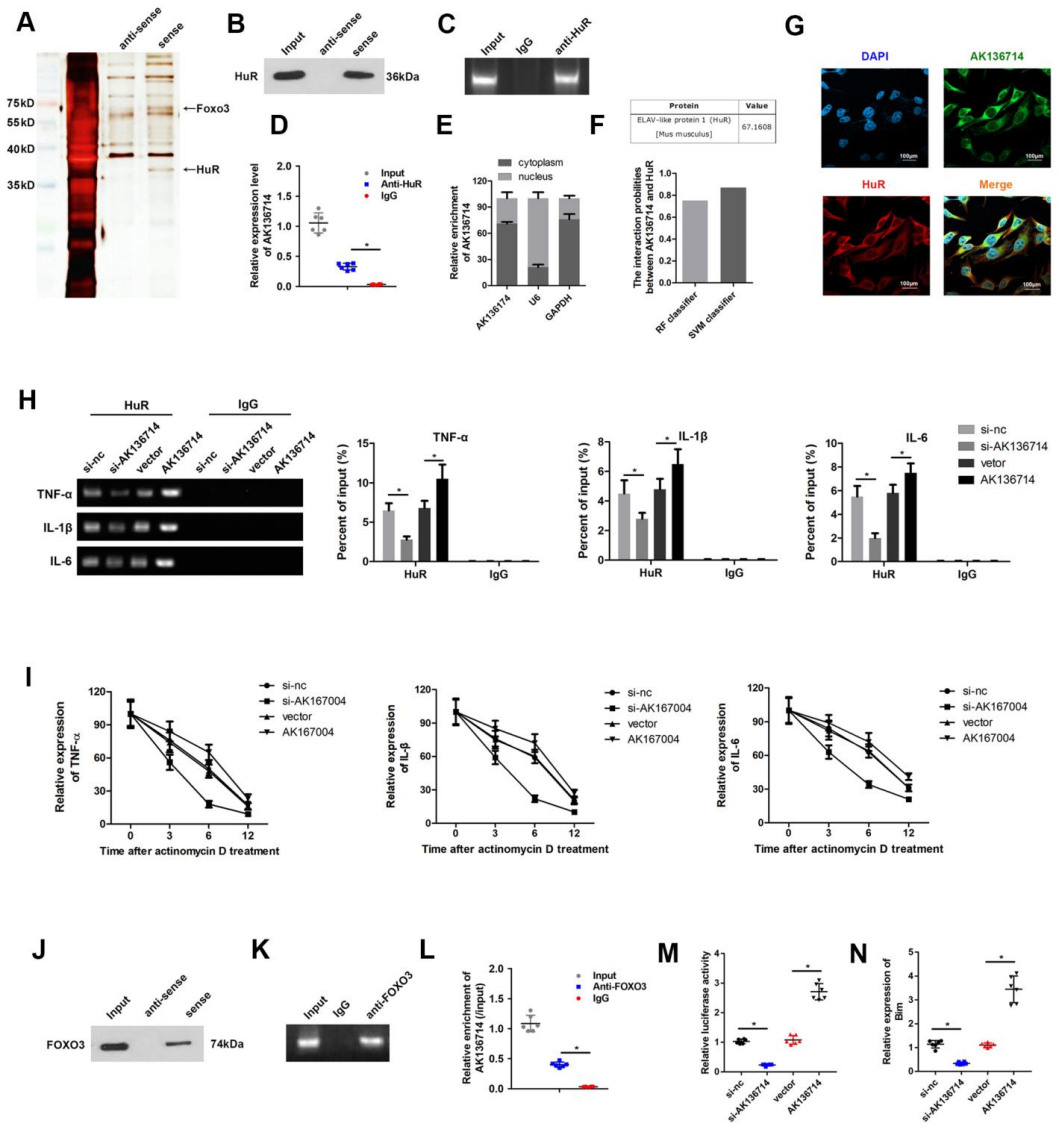
**AK136714 binds HuR and FOXO3.** (**A**) RNA pull-down, silver staining, and mass spectrometry analysis were performed to identify specific proteins that bind with AK136714. (**B**) RNA pull-down was used to verify that AK136714 bind to HuR. (**C**, **D**) RIP experiments verified that AK136714 binds HuR. (**E**) qPCR was used to detect nuclear AK136714 expression. (**F**) Bioinformatics analysis was performed to predict the possibility of the interaction between HuR and AK136714. (**G**) Immunofluorescence was performed to detect the colocalization of AK136714 and HuR. (**H**) Detection of inflammatory factors (TNF-α, IL-1β and IL-16) by RT-PCR after AK136714 overexpression or knockdown. (**I**) After inhibiting transcription with actinomycin D, the mRNA expression of each inflammatory factor was detected at different times by qPCR. (**J**) RNA pull-down was used to verify that AK136714 binds to FOXO3. (**K**, **L**) RIP experiments verified that AK136714 binds with FOXO3. (**M**) Luciferase assay was carried out to detect whether AK136714 silencing or overexpression altered the stimulating effect of FOXO3 on Bim. (**N**) qPCR was used to assess Bim levels after overexpression or knockdown of AK136714. (n=6, *P < 0.05, **P < 0.01).

We used RNA pull-down, silver staining, and mass spectrometry to analyze the proteins that interacted with AK136714, FOXO3 and HuR (Figure 5A). RNA pull-down confirmed that AK136714 was able to bind HuR (Figure 5B). RIP experiments further verified that AK136714 was able to bind HuR (Figure 5C, 5D). We found that AK136714 was mainly expressed in the cytoplasm (Figure 5E). Bioinformatics analysis also correlated AK136714 with HuR (Figure 5F). Cell immunofluorescence colocalization experiments showed that AK136714 was able to colocalize with HuR (Figure 5G). After AK136714 overexpression or suppression, RIP assay was performed and RT-PCR was used to detect the expression of inflammatory factors (TNF-α, IL-1β and IL-16) in the product pulled down by anti-HuR antibody. AK136714 inhibition reduces the binding of HuR to inflammatory factors (Figure 5H).

After using actinomycin to inhibit transcription, the mRNA levels of each inflammatory factor was detected at different times (3 hr, 6 hr, 12 hr), which showed that AK136714 promoted mRNA stability (Figure 5I). RNA pull-down experiments and RIP experiments also verified that AK136714 was binding to FOXO3 (Figure 5J–5L). We explored whether AK136714 could bind with FOXO3 and modulate the transcription of Bim. The luciferase assay results indicated that AK136714 overexpression promoted Bim transcription, while AK136714 silencing inhibited Bim transcription (Figure 5M). qPCR was used to evaluate the mRNA expression of Bim, which indicated that AK136714 promoted the expression of Bim while AK136714 knockdown inhibited Bim expression (Figure 5N).

### AK136714 inhibits endothelial cell apoptosis by binding to FOXO3

**Figure 6:**
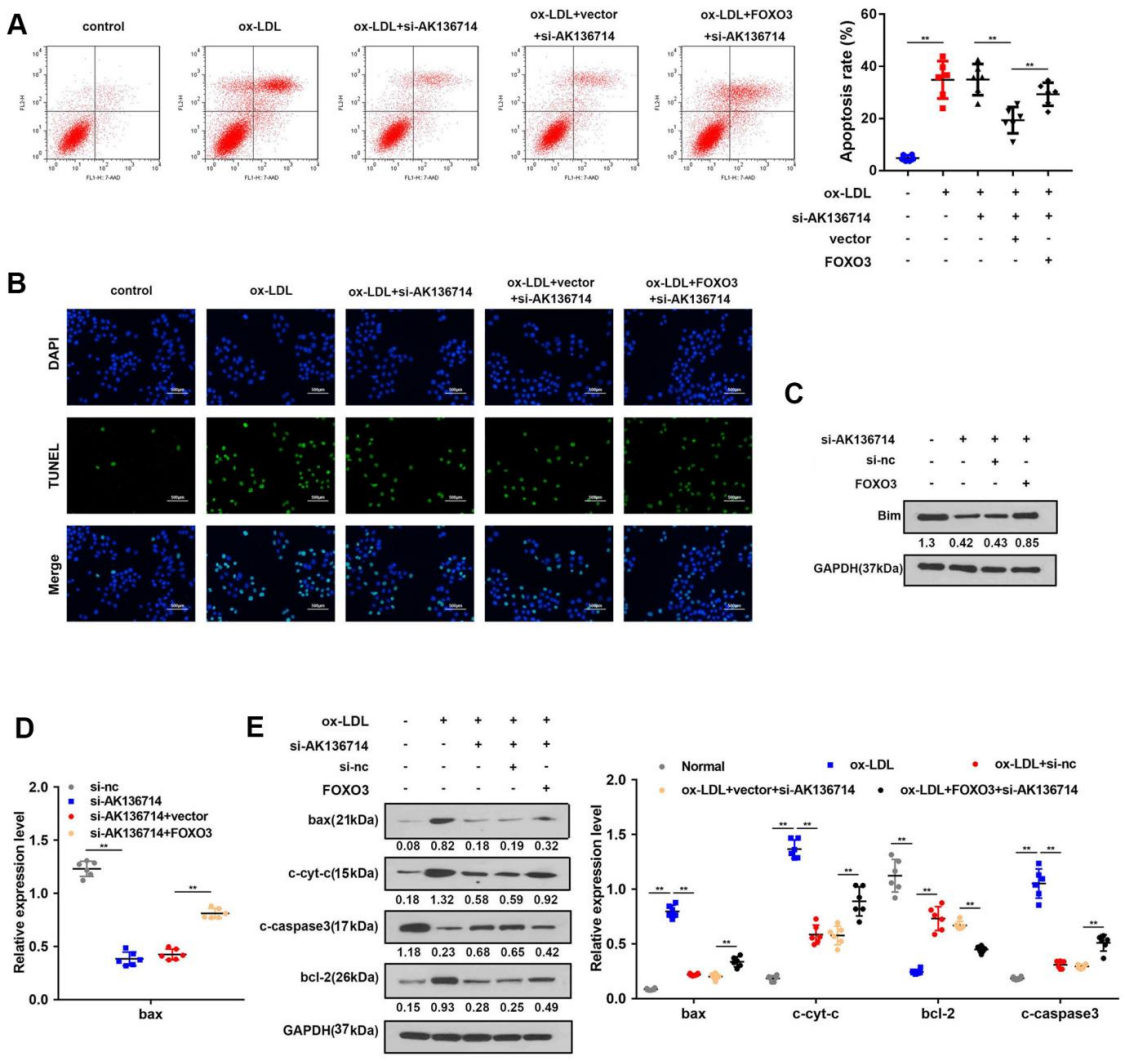
**AK136714 inhibits endothelial cell apoptosis by binding to FOXO3.** (**A**) Flow cytometry was used to analyze the apoptosis of endothelial cells. (**B**) TUNEL staining was used to analyze the apoptosis of endothelial cells. (**C**) Western blot was used to detect Bim expression in each group. (**D**) qPCR was performed to evaluate the expression of bax. (**E**) Western blot for detection of apoptotic protein expression in each group (n=6, *P < 0.05, **P < 0.01).

As Bim is a promoter of apoptosis, we investigated the effects of AK136714 and FOXO3 on endothelial cell apoptosis. Flow cytometry detection of apoptosis in each group showed that ox-LDL can induce apoptosis of endothelial cells, while silencing of AK136714 can inhibit endothelial cell apoptosis. FOXO3 can reverse the inhibitory effect of AK136714 (Figure 6A). The TUNEL results were consistent with those of flow cytometry (Figure 6B). Western blot showed that AK136714 silencing can inhibit the expression of Bim. FOXO3 overexpression reversed this effect of AK136714 (Figure 6C). qPCR results indicated that AK136714 knockdown inhibited the expression of Bax and FOXO3 can reverse this effect (Figure 6D). Western blot detection of the expression of apoptotic proteins in each group showed that ox-LDL increased the expression of apoptosis-related proteins Bax, c-cyt-c, and c-caspase3, while the expression of anti-apoptotic protein Bcl-2 decreased. Silencing of AK136714 inhibited the expression of Bax, c-cyt-c, c-caspase3, and promoted that of bcl-2. FOXO3 can reverse the anti-apoptotic effect of AK136714 knockdown (Figure 6E).

## DISCUSSION

Lipid metabolism disorders, endothelial injury theory, inflammatory response theory, and cerebral lymphatic drainage disorder all promote cerebral arteriosclerosis [16–21]. In cardiovascular disease, lncRNA expression can promote the occurrence and development of atherosclerosis by stimulating endothelial dysfunction, smooth muscle cell proliferation, foam cell formation, and lipid metabolism [22, 23]. We found that AK136714 silencing can inhibit inflammatory response and apoptosis of endothelial cell.

The intima of arterial vessels is an important barrier to regulate the material exchange between tissue and blood. A variety of factors can stimulate endothelial cells to malfunction and exfoliate, thereby diminishing the integrity and permeability of the intima. We found that AK136714 silencing can protect the endothelial barrier, which indicates that AK136714 silencing can protect endothelial cells and further inhibit the formation of atherosclerotic plaques. Atherosclerosis is not a simple lipid deposition disease in the blood vessel wall, but a process of chronic low-grade inflammation and oxidative stress. As atherosclerosis progresses, inflammatory cells and macrophages phagocytosing Ox-LDL increase and produce tumor necrosis factor-α (TNF-α), interleukin-8 (IL-8), interleukin-1 (IL-1), and other important inflammatory factors that further aggravate atherosclerosis. We found that AK136714 silencing can inhibit the release of inflammatory factors TNFα, IL-1β, and IL-6.

FOXO transcription factors can inhibit the proliferation of vascular smooth muscle cells (VSMCs), the migration of endothelial cells, the proliferation and hypertrophy of cardiomyocytes, and antioxidant stress [24–26]. AKT can phosphorylate FOXO3, inhibit VSMCs apoptosis at the plaque sites, protect the stability of the plaque, and reduce the incidence of cardiovascular accidents. The activation of FOXO3 can increase the apoptosis of SMCs and decrease plaque stability [27]. Tsuchiya et al. found that knocking out FOXO transcription factors in endothelial cells can increase NO synthesis, reduce inflammation and oxidative stress, and prevent atherosclerosis in mice with low-density lipoprotein receptor deficiency [28]. FOXO3 has dual effects of protecting and damaging cardiovascular function. We found that AK136714 binds with FOXO3 and activates the transcription of pro-apoptotic gene, *Bim*. ChIP or EMSA assays could confirm the interaction between FOXO3 and Bim.

We found a novel lncRNA, AK136714, which is increased in the plaque tissue and blood of atherosclerotic mice. Silencing of AK136714 inhibited inflammation and apoptosis of endothelial cells, and increased endothelial cell permeability.

## MATERIALS AND METHODS

### Main materials and reagents

(21 ± 1.8) g Male C57BL/6J mice and C57BL/6J, ApoE^-/-^mice were purchased from the Institute of Basic Studies, Peking Union Medical College. Human umbilical vein endothelial cells (HUVECs) were purchased from ATCC cell bank in the U.S. DMEM high glucose medium dry powder and fetal bovine serum (FBS) were purchased from Gibco in the U.S. Dimethyl sulfoxide (DMSO) was purchased from Sigma, USA. Oxidized low density lipoprotein (ox-LDL) was purchased from Guangzhou Yiyuan Biotechnologies. TNF-a detection kit was purchased from American Adipo Bioscience Company. BCA kit was purchased from Shanghai Biyuntian Biotechnology Company.

### Establishment of an atherosclerotic animal model

The mice were housed in an environment with light and dark (12h: 12h), temperature (22° C), humidity 50%. The ApoE mice in atherosclerosis group were fed high-fat diets (21% fat and 0.15% cholesterol) at 4 weeks of age. C57BL/6J was used as the normal control group and fed an ordinary diet (4% fat and 0% cholesterol). After 12 weeks, the mice were sacrificed and the aortas were removed for experiments.

### Cell culture

HUVECs were used after 2-3 passages. After HUVECs were resuscitated, they were cultured in a high-glucose DMEM medium (containing 10% FBS, 1% penicillin) in a 37° C, 5% CO_2_ incubator. When the cell density reached 80%, the cells were digested and passaged with 0.25% trypsin. HUVECs were stimulated with ox-LDL (50 μg/ml) for subsequent cell experiments.

### Cell lentiviral vector construction and cell transfection grouping

Adenovirus silencing AK136714 and overexpressing FOXO3 were established by Hanbio (Shanghai, China). Endothelial cells were cultured for 24 hours under stimulation with ox-LDL (50 mg/dL), and an atheroma model of the cells was cultured. After the endothelial cells attained 50% to 70% density, lentiviral stock solution (3.5x10^7^ viral particles) was added, and Pollybrene reagent (Beyotime, Shanghai, China) was added to increase the infection efficiency. After 24 hours, the solution was changed and the new culture medium was added.

### Transfected cells

Before transfection, HUVECs in the logarithmic growth phase were digested and seeded at 1.0x10^6^ cells per well in 6-well plates. The medium (containing 10% FBS) was added, then the plates were shaken and placed in a 37° C incubator. Cell transfection was performed according to the manufacturer’s instructions (Beyotime, Shanghai, China). The medium was replaced after 3 to 4 hours, and then the cells were cultured in a CO_2_ incubator for subsequent experiments.

### Determination of TNFα, IL-1β and IL-6

The cell culture supernatant was centrifuged in a 4° C centrifuge at 2000 rpm for 5 min, and the supernatant was collected and stored at -80° C for testing. The experiment was performed according to the instructions of the TNF-α, IL-1β and IL-6 detection kit. The absorbance value of the sample was measured, and the corresponding concentration was calculated according to the standard curve.

### Western blot

Total protein was extracted using a commercial kit according to the manufacturer’s instructions (Beyotime, Shanghai, China). The protein concentration was determined in a Nanodrop 2000 system (Thermo Fisher, USA). SDS-PAGE gel was prepared, 40 μg protein was loaded for electrophoresis, then transferred to a PVDF membrane. The blots were then blocked in 5% skim milk at room temperature for 2 hr. The blots incubated with the primary antibody (1: 1000) in a shaker over night at 4° C. The blots were then incubated with secondary antibody (1: 5000) at room temperature for 2 hr. The blots were visualized by ECL chemiluminescence, and the gray value of each band was analyzed with QuantityOne software. GAPDH was used as the internal control.

### Pathological and morphological changes of rat aorta detected by HE staining

After being thoroughly anesthetized, the rat’s thorax was exposed. The aorta was quickly excised, starting at the beginning of the aorta, through the bifurcation of the aortic arch to the descending aorta, and 2 to 3 cm of the thoracic aorta. We included the myocardial tissue at the root of the aorta, washed with ice, and then washed the aorta. The root and the aortic arch bifurcation vessels were immediately fixed in a 4% neutral formaldehyde solution, dehydrated by 75% ethanol, placed in xylene and transparent, and then immersed in wax. The embedded wax block was fixed on a microtome and sliced continuously with a thickness of 5 μm. The slices were flattened and dried on a glass slide and stained with HE. The aorta HE stained sections of each group were assessed under the light microscope.

### PCR

Trizol reagent was used to extract total RNA from serum and brain tissues. The purity and concentration of RNA were detected by UV spectrophotometer. Reverse transcription was performed according to the Ribo™ mRNA/lncRNA qRT-PCR Kit instructions, followed by qPCR. PCR reaction conditions: pre-denaturation at 94° C for 5 min, then 40 cycles of denaturation at 95° C for 10 s, annealing at 60° C for 30 s, and extension at 72° C for 30 s. U6 was used as the internal reference, and the results were analyzed by 2^-ΔΔCt^ method.

### Flow cytometry to detect apoptosis

After treating the cells according to the requirements of each group, the cells were trypsinized, collected, and then washed twice with pre-chilled PBS at 4° C. After centrifugation, the PBS supernatant was discarded, and the cells were resuspended with buffer. After 5 μl of NanoVin-FITC and 1 μl of 0.1 g/l PI double fluorescent labeling was added, flow cytometry was used to detect cell apoptosis, which were analyzed using the Flowjo software.

### RNA pull-down

The biotinylated probe of AK136714 and the control probe were synthesized by Sangon Biotech (Shanghai, China). Probe-coated beads were generated by co-incubating the probe with streptavidin-coated beads (Invitrogen, CA, USA) at 25° C for 2 h. Cells were lysed to extract total protein. After pretreatment of magnetic beads, RNA and beads were mixed. After separation, western blot and mass spectrometry were used to verify the downstream binding protein of AK136714.

### Vascular oil red O staining

The specimen was removed from the formaldehyde solution, washed with running water for 15 minutes, had the outer membrane peeled off, then dipped in distilled water. After we added 40 mL distilled water to 60 mL of the stock solution, we let it stand for 10 minutes and then stained it for 3 hr. The specimen was soaked with 70% ethanol until the plaque was red and the background was white. Finally, the specimen was soaked with distilled water and immersed in the formaldehyde solution for storage.

### Statistical analysis

SPSS18.0 software was used for data analysis. Comparison between groups was performed by one-way analysis of variance and t test. The data were expressed by x ± SD, and P < 0.05 indicated a statistically significant difference.

### Data statement

Data are available from the corresponding author with reasonable request.
